# Moral distress - a threat to dementia care? A qualitative study of nursing staff members’ experiences in long-term care facilities

**DOI:** 10.1186/s12913-022-07695-y

**Published:** 2022-03-03

**Authors:** May Helen Midtbust, Eva Gjengedal, Rigmor Einang Alnes

**Affiliations:** 1 Norwegian University of Science and Technology, Faculty of Medicine and Health Sciences,, Department for Health Sciences in Aalesund, Box 1517, 6025 Aalesund, NO Norway; 2 University of Bergen, Department of Global Public Health and Primary Care, Box 7804, 5020 Bergen, Norway

**Keywords:** Moral distress, Ethical conflicts, Dementia, Palliative care, End-of-life care, Long-term care facilities, Qualitative methods

## Abstract

**Background:**

Dementia is a public health priority worldwide due to its rapidly increasing prevalence and poses challenges with regard to providing proper care, including end-of-life care. This study is part of a research project about nursing staff members’ experiences with providing palliative care for people with severe dementia in long-term care facilities. In an earlier study, we found that structural barriers that complicated the provision of palliative care led to moral distress among nursing staff. In this study, we performed a secondary analysis of the same data set to gain a deeper understanding of nursing staff members experiences of moral distress while providing palliative care for residents with severe dementia in long-term care facilities.

**Methods:**

A qualitative, descriptive design was used. Data were collected during in-depth interviews with 20 nursing staff members from four Norwegian long-term care facilities. Content previously identified as moral distress was reanalysed by thematic text analysis, as described by Braun and Clarke, to gain a deeper understanding of the phenomenon.

**Results:**

The nursing staff members’ experiences of moral distress were generally of two types: those in which nursing staff members felt pressured to provide futile end-of-life treatment and those in which they felt that they had been prevented from providing necessary care and treatment.

**Conclusion:**

The findings indicate that nursing staff members’ experiences of moral distress were related to institutional constraints such as time limitations and challenging prioritizations, but they were more often related to value conflicts. Nursing staff members experienced moral distress when they felt obligated to provide care and treatment to residents with severe dementia that conflicted with their own values and knowledge about good palliative care. Both education interventions focused on improving nursing staff members’ skills regarding communication, ethical judgement and coping strategies; in addition, supportive and responsive leadership may have significant value with regard to reducing moral distress. Our findings indicate a need for further research on interventions that can support nursing staff members dealing with ethical conflicts in providing palliative care to residents with dementia.

## Introduction

This study focuses on the ethical challenges leading to moral distress experienced by nursing staff members providing palliative care to people with severe dementia in long-term care facilities. Moral distress has been widely explored in a variety of settings in which nurses provide acute or intensive care [1–4]. Situations that can lead to moral distress among nursing staff in long-term care facility settings have been less studied. This is particularly true in the context of the provision of palliative care to residents with severe dementia.

Dementia is a public health priority worldwide due to its rapidly increasing prevalence and poses challenges with regard to providing proper care, including end-of-life care [5–7]. Previous studies on palliative care for people with dementia have often focused on interventions to improve end-of-life practices in long-term care facilities [8–10] but have to only a small extent explored how nursing staff experience the challenges of caring for residents with severe dementia.

A literature review on the ethical issues experienced by nursing staff in long-term care facilities showed that challenges associated with communication, the lack of resources and the quality of the care provided were associated with staff burnout and moral distress [11]. In some high-income countries, approximately 50 to 80% of the residents in long-term care facilities have dementia [5, 12], and previously published studies confirm that caring for people with dementia in such facilities is related to moral distress among the nursing staff [13–16]. A prevalence study revealed that nursing staff members reported experiencing moral distress at least daily or weekly and pointed out that moral distress is a prevalent experience among staff who care for people living with dementia. The consequences of moral distress, such as frustration, physical exhaustion, a feeling of being emotionally drained and a feeling of powerlessness, were also reported as occurring at least weekly in nearly half of the participants [13]. Powerlessness was often associated with decisions made by relatives and GPs about interventions that nursing staff perceived to be futile and the cause of more suffering for the resident [13, 14, 16]. Other studies have examined sources of moral distress in nursing staff members providing care to residents with dementia, and a lack of resources that led to a poor quality of care was reported as a main source of moral distress. Time constraints and a working culture characterized by business concerns and challenging prioritizations especially affected the weakest bedridden residents with severe dementia, and nursing staff felt guilty for spending too little time caring for each individual [14, 15, 17]. Additionally, conflicting expectations with regard to care meant that nursing staff members felt bound to provide care that conflicted with their own beliefs and knowledge, which was connected to their experience of moral distress.

Previous research shows that moral distress is prevalent among nursing staff who provide dementia care in long-term care facilities. Moral distress related to end-of-life care has, however, rarely been a research topic. In light of the increasing occurrence of residents with dementia dying in long-term care facilities, there is a need for more research focusing on the challenges leading to moral distress among nursing staff members caring for residents with severe dementia in their final phase of life. In an earlier study*,* we found that nursing staff members experienced structural barriers that complicated the provision of palliative care to residents with severe dementia at the end of their lives in long-term care facilities and led to moral distress among nursing staff [18]. In the present study, we performed a secondary analysis of the same data set to gain a deeper understanding of nursing staff members’ experience of moral distress in these situations.

### Moral distress

Alongside the increasing body of research on moral distress, there has been growing interest in the way in which the concept of moral distress has been defined and understood [1, 19–22]. The concept was first defined by the philosopher Andrew Jameton in 1984: “Moral distress arises when one knows the thing to do, but institutional constraints make it nearly impossible to pursue the right course of action” [23]. He sought to capture what he observed as an emerging feature of the professional role of nursing – that nurses were unable to act in a way that was consistent with their ethical values because of institutional obstacles [19, 24]. Later empirical research on nurses’ experiences with moral distress led to the enhancement of the original definition [25–27], and in 1993, Jameton expanded his definition by distinguishing between two forms of distress, namely, initial and reactive: “Initial distress involves the feeling of frustration, anger, and anxiety people experience when faced with institutional obstacles and conflict with others about values. Reactive distress is the distress that people feel when they do not act upon their initial distress” [28].

**Fig. 1:**
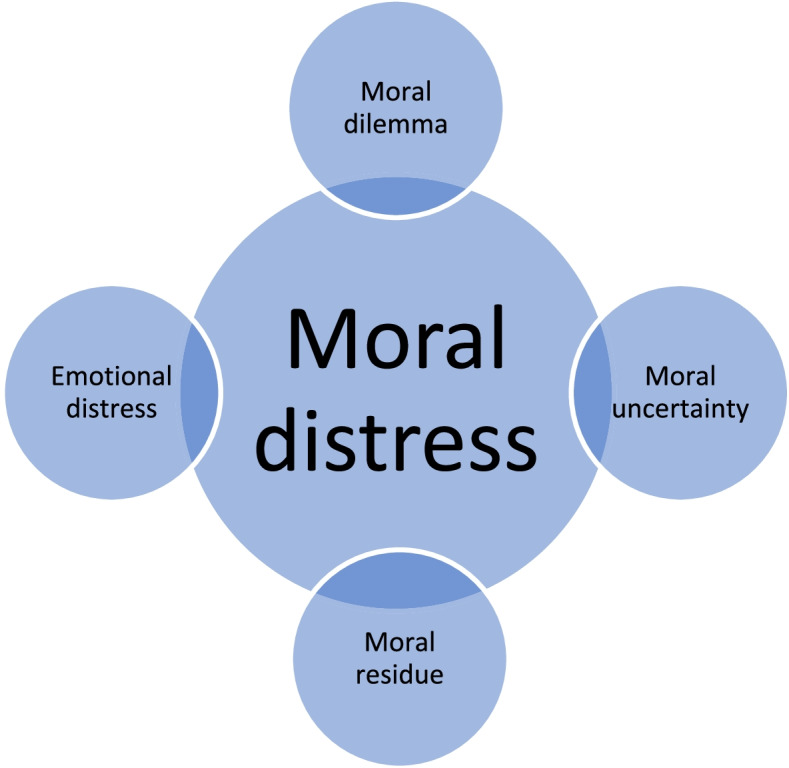
Moral distress: connection with related constructs

In this new definition, he emphasized the psycho-emotional responses of not acting in a morally appropriate way. Reactive distress is also referred to as “moral residue” and was later recognized as a concept that is different from, yet related to, moral distress [21, 29]. In addition to moral residue, other constructs, such as emotional distress, moral uncertainty and moral dilemmas, can all be associated with and partially overlap with moral distress. According to Jameton, the main features that distinguish moral distress from these constructs are the concurrent feeling of being constrained from taking the ethically appropriate action “when one knows the thing to”. Moral judgement and institutional constraints thus appear to be necessary and sufficient conditions for Jameton’s definition of moral distress and differentiate moral distress from other constructs, as shown in Fig. 1 [1, 19–22].

## Methods

### Design

In a previous study with a qualitative and descriptive design, we interviewed 20 nursing staff members to gain insight into their experiences of providing palliative care to residents with severe dementia in long-term care facilities [18]. Although these interviews did not focus on moral distress, we found that this information was nonetheless provided. In the present study, we therefore sought answers to new research questions about moral distress to gain a deeper understanding of nursing staff members’ experience of moral distress when providing palliative care for residents with severe dementia in long-term care facilities.

### Participants and recruitment

The management teams of four long-term care facilities in three diverse municipalities in mid-Norway were asked to recruit nursing staff members for the study. To ensure some variation in the sample, two long-term care facilities in a mid-sized city and two in smaller municipalities were randomly selected. The manager of each unit gave the nursing staff verbal and written information about the purpose of the study. Those who wanted to participate could either contact the first author or the manager directly. No one on the research team had any relations with the participants or the units they worked at.

The size of the long-term care facilities was quite similar, with 48 to 78 beds, and they included short- and long-term units. Three of them had sheltered units for residents with a dementia diagnosis with approximately six to 12 residents. Both enrolled nurses (EN) and registered nurses (RN) were included in the study. Although RNs bear the main responsibility for providing palliative care in long-term care facilities, ENs play an important role in direct patient care. Seven ENs and 13 RNs, five from each long-term care facility, participated in the study. The participants, all of whom were women, were employed in half-time to full-time positions. The average age was 43 years (range 28–63), and they had three to 40 years (average 18) of experience working with residents with dementia. Details of the participants are illustrated in Table 1.

**Table 1:** Details of participants

Participants (all women)	Education Registered nurse (RN) Enrolled nurse (EN)	Unit	Age	Position	Work experience (years)	Long-term care facility (LTCF)
P1	RN	Short-term	34	100%	10	LTCF 1
P2	RN	Sheltered	50	75%	25	LTCF 1
P3	EN	Sheltered	42	75%	20	LTCF 1
P4	RN	Long-term	31	100%	6	LTCF 1
P5	RN	Long-term	33	100%	12	LTCF 1
P6	EN	Long-term	41	67%	13	LTCF 2
P7	RN	Long-term	40	100%	4	LTCF 2
P8	RN	Short-term	33	80%	4	LTCF 2
P9	EN	Short-term	63	50%	40	LTCF 2
P10	RN	Short-term	52	100%	30	LTCF 2
P11	EN	Sheltered	56	100%	14	LTCF 3
P12	RN	Long-term	28	80%	5	LTCF 3
P13	RN	Sheltered	61	80%	14	LTCF 3
P14	RN	Long-term	34	80%	3	LTCF 3
P15	EN	Sheltered	44	70%	14	LTCF 3
P16	EN	Long-term	51	80%	29	LTCF 4
P17	RN	Sheltered	46	100%	20	LTCF 4
P18	RN	Long-term	34	100%	11	LTCF 4
P19	RN	Short-term	45	100%	4	LTCF 4
P20	EN	Sheltered	53	88%	30	LTCF 4

### Data collection

The data collection method in the study was in-depth interviews with nursing staff members working with residents with dementia in long-term care facilities. The first author conducted all the in-depth interviews. She is a nurse and has experience from the field of dementia care and qualitive research. The two other authors (EG and REA) are nurses and experienced qualitative researchers.

During the interview, the first author searched for varied and rich descriptions of nursing staff members’ experiences. The supportive dialogue involved asking open-ended questions, giving the informants time and space to talk without interruption, listening actively, and asking for further explanation when appropriate. A semistructured interview guide was used to help balance openness and focus during the interview [30, 31]. The interviews started with the following question: What are your experiences with providing palliative care to residents with severe dementia? All the interviews were conducted in suitable meeting rooms at long-term care facilities and lasted approximately 60 min. The interviews were recorded and transcribed verbatim by the first author.

### Data analysis

The original data set was reanalysed by asking new research questions about nursing staff members’ experience of moral distress when providing palliative care to residents with severe dementia in long-term care facilities. In the analyses, we used a stepwise method inspired by thematic text analysis, as described by Braun and Clarke [32]. First, the interview transcript was read several times to enable the researcher to become acquainted with the content. In the next step, all text about moral distress, as defined by Jameton [23, 28], was marked and separated from the rest of the material. The material about moral distress was then organized by generating and applying initial codes. This process continued until all relevant material was coded. Furthermore, themes were identified by grouping related codes. In the last step, preliminary themes were discussed and adjusted until the research group agreed on the final themes that addressed the purpose of the study. All the authors participated in the analysis. An illustration of the analysis process is presented in Table 2.

**Table 2:** Illustration of the analytical steps

Quotes	Initial codes	Preliminary themes	Final themes
“Next of kin want intravenous treatment for every infection, but they do not see how demanding it is for the resident, as they are not present all the time” (P4) “It is quite demanding to perform (intravenous treatment)– it goes so much against what I should do for the resident, but then you do it for their next of kin” (P17)	Next of kin and nursing staff have different opinions about treatment levels Disagreements with next of kin about treatment are highly demanding experiences for nursing staff	Nursing staff feel forced to give painful treatment Nursing staff feel forced by next of kin to provide futile treatment	Feeling compelled to provide futile end-of-life care
“You are left quite helpless. When your offer is refused when they just reject you. Then, how are you going to help? “(P9) “It is an ethical dilemma when you see how they (the residents) are not comfortable.” P19	Nursing staff feel helpless when “care” is rejected, and they are not able to help the resident with pain relief Provision of pain relief is morally difficult when residents are unable to understand why such measures are implemented	Nursing staff are rejected from helping the residents with pain relief Providing pain relief against the resident’s will is morally difficult	Being prevented from providing necessary pain relief at the end of life
“I feel bad, but I truly do not have a choice. Because there are so many requiring my assistance” P12 “You have to postpone palliative care until the definite end; it is only at the very end of their life they get that hand to hold” P16	Nursing staff feel bad because they have to little time to each resident Nursing staff experience lack of recourses make them do hard prisonizations	Nursing staff feel torn between spending time with each resident and the pressure of spending time with all the residents Time constrains require hard prioritizations	Being exposed to time constraints and challenging prioritizations

## Results

The nursing staff members’ experiences of moral distress could be categorized into two groups. In some cases, they felt pressured to provide futile end-of-life treatment. In other cases, they felt prevented from providing necessary care and treatment. Nursing staff members reported that many next of kin were unable to realize or accept how ill their close family members were, often demanding treatment that caused more suffering than relief, from the nursing staff members’ point of view. Moral challenges also arose when nursing staff were obstructed from attempting to provide necessary pain relief in end-of-life care and when they were exposed to time constraints and challenging prioritizations.

### Feeling compelled to provide futile end-of-life care

All participants had experienced feeling pressured to provide futile treatment to residents with severe dementia. In various ways, the participants described how some next of kin demanded that nursing staff administer treatments that the nursing staff members believed would lead to more suffering than relief. They reported that many next of kin were unable to realize or accept that their close family members were gravely ill and requested treatment that nursing staff members knew would be painful. One nurse said, “*Next of kin want intravenous treatment for every infection, but they do not see how demanding it is for the resident, as they are not present all the time*” (P4). The resident might appear satisfied and pain-free when next of kin were present, but the nursing staff observed increased pain, difficulties eating and other afflictions, none of which the next of kin observed during their occasional visits. Additional difficulties may arise when the next of kin resides far away, and they have not seen their family member for an extended period of time. One nurse expressed it as follows: *“I have experienced them entering the patient’s room, greeting their mother or father for the first time in maybe a year, and then they are shocked by what they see” (P5).* These next of kin have not seen the gradual deterioration, which may have been observed by other family members, and disagreements may arise between them. These situations may be difficult for all parties, including the nursing staff, who were under the impression that the situation was clear and that there was agreement about the resident’s trajectory.

Disagreements with next of kin about what is best for a resident are highly demanding experiences for nursing staff. One nurse told a story about a particularly difficult situation in which she felt forced to administer painful and useless treatment to a person with severe dementia at the end of life. The resident became increasingly ill and developed one infection after another, and the nursing staff members observed that the resident’s life was ending. Nursing staff in the ward reported that the next of kin were unable to accept that their close family member was severely ill and requested continued intravenous treatment with antibiotics despite advice to the contrary from both the physician and the nurse. The nursing staff felt that the treatment imposed a greater burden on the resident rather than providing relief and that treatment continued only to appease the demands of the next of kin and not out of concern for the resident’s well-being.

Such demands from next of kin were highlighted as particularly difficult when related to residents with severe dementia who were no longer able to verbally express their needs. One nurse said, *“How often should you inject a person who does not understand why you are doing it, and you may have to restrain the resident a little to administer the injection in the right way. It is hard to defend when the resident expresses severe discomfort from these injections”* (P4).

Multiple participants described such situations as ethical dilemmas in which they were prevented from providing good palliative care at the end of life. They experienced it as agonizing to be compelled to provide treatment that focused more on prolonging life than increasing quality of life. One nurse shared her experiences with what she called “extreme” cases related to the intravenous treatment of persons with severe dementia: “*It is quite demanding to perform – it goes so much against what I should do for the resident, but then you do it for their next of kin. Because you cannot truly defend it professionally. Therefore, this is a constant dilemma”* (P17).

### Being prevented from providing necessary pain relief at the end of life

Being prevented from providing treatment and sufficient pain relief were moral challenges experienced by nursing staff. They reported how a lack of examination and treatment meant that some residents did not receive sufficient pain relief. One nurse shared her experiences in a difficult situation in which nursing staff had observed that a resident was in severe pain for an extended time. The nurse conferred with a physician and argued that the resident should be examined at a hospital, as the long-term care facility staff were unable to sufficiently relieve her pain. The nursing staff members expressed the despair they experienced when the resident was not scheduled for examination despite their frequent appeals to physicians. Eventually, an examination was performed, and the cause of the pain was found to be severe cancer. The nurse said that she experienced feelings of being too late with regard to providing pain relief that weighed heavily on her mind. The result was a very grim situation in which both nursing staff and next of kin knew that the resident was in severe pain until she died. The nurse related: *“We did everything in our power to relieve her pain, but afterwards we all felt we had been unable to give sufficient pain relief” (P2).*

Nursing staff also reported that some next of kin fear that their loved ones will receive excessive medication towards end of life and that many are particularly afraid of excessive morphine administration. Several participants expressed understanding the next of kin wanting what is best for their loved ones but also feeling dispirited at not being given the opportunity to provide sufficient pain relief to residents at the end of their life. To illustrate how demanding such situations can be, one nurse told a story about how painful it was to feel prevented from providing good palliative care to a resident. This nurse works in a specialized ward for persons suffering from dementia and has extensive experience as well as additional education regarding palliative care and treatment. During one evening shift, she was also responsible for a somatic long-term care ward. She was summoned because a resident suffering from dementia was terminally ill and suffering from pain. Prior to entering the patient’s room, she was informed that the next of kin were very sceptical of morphine and that an agreement to not administer morphine had been made with a physician. When the nurse saw the resident, her first impulse was to administer morphine. However, because the next of kin had discussed the issue with both physicians and nursing staff assigned to the current ward, she felt compelled to abstain from administering morphine. She said, *“This turned out to be an agonizing situation where I felt unable to provide sufficient care for the resident” (P13).* She explained afterwards that she was left with a feeling of having been prevented from helping and providing palliative care at the end of life to the resident and a sense that the demands from the next of kin were more important than her professional assessment as a nurse.

Being prevented from providing necessary pain relief at the end of life also exacerbated nursing staff members’ experiences of feeling rejected and not being given the opportunity to care for a resident in pain. Nursing staff members frequently reported finding themselves in highly demanding situations in which they were trying to provide care, but the residents responded by rejecting and challenging them. One enrolled nurse said, *“You are left quite helpless. When your offer is refused, when they just reject you. Then, how are you going to help?” (P9).*

The feeling of having come close to committing assault when required to use force and act in ways that the resident clearly opposes were described as painful experiences by several participants. One nurse shared her experience in a situation in which she truly felt she had failed as a nurse. An older woman with severe dementia behaved aggressively and dismissively towards the nursing staff. They observed that the woman’s condition gradually worsened, and she seemed increasingly affected by pain. In the final days of her life, she suffered from multiple infections that did not respond to treatment, and she rejected all attempts at treatment and palliative care. The nurse related that the situation was very demanding. She wanted very badly to help the woman feel as comfortable as possible. When she tried to help, she was beaten and verbally assaulted. The nurse described this as imposing a major psychological strain and reported how she was unable to provide care regardless of what she did. Multiple drugs were considered, but eventually, the resident was practically sedated with morphine. The nurse said: *“It was very hard. You feel inadequate and almost unable to help the resident in any other way than to sedate her with morphine” (P4).*

The provision of pain relief to residents suffering from severe dementia who were unable to comprehend why such measures were being implemented was commonly experienced as a major challenge. Nursing staff explained how these situations typically arose when the residents refused to take their pills and how administering pain relief via injections felt like assault when it was performed against the resident’s will. One nurse mentioned not only how she frequently resorted to using analgesic patches but also how morally difficult she found it to practically deceive the resident. She said, *“It is an ethical dilemma when you see how they [the residents] are not comfortable. In addition, it is not just pain relief, it also sedation. It is hard to decide how to approach these situations because you want them to be comfortable”* (P19).

### Being exposed to time constraints and challenging prioritizations

Nursing staff constantly experience increased demands, as the residents admitted to long-term care facilities are severely ill and have comprehensive care needs. Resource allocation does not meet these demands. Despite this, staff are required to reduce spending. One enrolled nurse explained, *“They are cutting back on everything. You must do dishes, laundry, you are doing everything. It is not just care you are required to provide… We have too few nursing staff around patients”* (P16).

The participants talked about time constraints and challenging prioritizations that left bedridden residents with severe dementia particularly vulnerable. They experienced feeling torn between spending time with each resident and the pressure of spending time with all the residents. The time constraints resulted in feelings of guilt and the inability to provide the palliative care they would like to be able to give. One nurse explained that she always felt guilty because she had too little time to provide quality care. She explained this by relating an example of a situation involving a meal during which she was supposed to provide guidance to residents enabling them to feed themselves, but a lack of time led to her feeding the residents instead. She said, *“I feel bad, but I truly do not have a choice. Because there are so many requiring my assistance” (P12).*

Even though the participants experienced hectic workdays characterized by time constraints and difficult prioritizations, they made the extra effort to ensure that the end of life was as pleasant as possible for residents and their next of kin. They reprioritized tasks and did their best to ensure that nursing staff were always present with dying residents. Despite nursing staff members’ efforts to provide a comfortable and dignified death, the nursing staff wished they had more time to devote to each patient earlier in the course of illness. The participants reported a general lack of the resources needed to provide palliative care to residents with severe dementia. For some patients, the final phase lasts for a month, while for others, it lasts only a few days. Scarce resources mean that nursing staff have to evaluate the condition of the residents and determine if they are truly dying before they reprioritize resources or hire nursing staff to stay with the dying resident. One enrolled nurse said, *“You have to postpone palliative care until the definite end; it is only at the very end of their life they get that hand to hold”* (P16).

## Discussion

The purpose of this study was to explore nursing staff members’ experiences of moral distress when providing palliative care to residents with severe dementia in long-term care facilities at the end of life. The major findings are that nursing staff members’ experiences of moral distress are related to situations in which the care and treatment provided caused the person with dementia to experience increased suffering. According to Jameton’s definitions [23, 28], nursing staff were exposed to moral distress because they were unable to act in a way that was consistent with their ethical values and beliefs regarding good palliative care because of institutional obstacles. In his first definition (1984), moral judgement and institutional constraints seem to be necessary and sufficient conditions for moral distress, but in his later definition (1993), he includes “conflict with others about values”. Institutional constraints, such as the lack of resources, that led to a poor quality of care were reported as the main source of moral distress among nursing staff caring for residents with dementia in earlier studies [14, 15, 17]. Additionally, our findings indicate that moral distress was related to institutional constraints, such as time constraints and challenging prioritizations, but that they were perhaps more often related to what Jameton described as “conflict with others about values”. The most prominent cause of moral distress was the nursing staff members’ experiences of feeling compelled to provide futile end-of-life treatment or feeling that they were prevented from providing necessary pain relief at the end of life. Such situations were often associated with demands from the next of kin for staff to provide care and treatment that, in their estimation, focused more on prolonging life than increasing quality of life. The line between palliative care and treatment that is considered futile may be blurred. The assessment of the appropriateness of a given treatment may depend on the staff members’ knowledge of palliative care and dementia [33] and their clinical experience, and there may be differences in values among professionals and between professionals and the next of kin. Additionally, previous research identified conflicting values with regard to care as a factor contributing to moral distress among nursing staff members working with residents with dementia. Moral distress was experienced when the nursing staff members felt bound to provide care that conflicted with their own beliefs and knowledge regarding what the resident might want or need  [13, 14, 16]. Conflicts between values pertaining to palliative care may be particularly difficult when the patient has severe dementia and is no longer able to verbally express themselves. Nursing staff in our study highlighted how difficult it was to feel pressured to give painful treatments to residents who expressed severe discomfort or to be prevented from helping either by the next of kin or the residents themselves.

In Jameton’s 1993 definition of moral distress, he included the psycho-emotional consequences of moral distress, such as feelings of frustration, anger, and anxiety [28]. In a literature review about moral distress in nursing in the general field of care for the elderly population, the results indicate that nurses are primarily affected in two ways. Moral distress may have consequences for themselves or others and consequences for the system [34]. In our study, the nursing staff described the consequences of moral distress for themselves, such as feelings of inadequateness, frustration, and powerlessness, when they were prevented from providing what they believed to be good palliative care. Similar findings were reported in a prevalence study of moral distress in dementia care in which nearly half of the participants reported feeling frustrated, physically exhausted, emotionally drained, and powerless at least weekly as a result of moral distress [13]. Nursing staff in our study also expressed feelings of guilt and a heavy conscience, which reflect the consequences experienced by others when time constraints meant that they were unable to spend time with vulnerable bedridden residents with severe dementia and when care and treatment caused increased suffering at the end of life. Previous research highlighted the risk of becoming callous, bitter, cynical, or frustrated as a consequence of being exposed to such moral distress over time. If nursing staff members cannot or choose not to discuss or act upon the problem causing moral distress, it can contribute to issues with quality of care and patient satisfaction [34]. This may be in line with what Jameton describes as moral residue, which is both a contributor to and a consequence of moral distress (see Fig. 1) [21, 28, 29]. The residual effect may also create consequences for the system, such as issues of nurse retention and staff shortages [34]. Poor staffing levels, high turnover and demanding workloads are prevalent in long-term care facilities [17, 35, 36]. Hence, moral distress may have implications for recruitment to the profession [34].

How can moral distress be prevented? One strategy may be to increase nursing staff members’ education regarding communication, ethical judgement and coping strategies [34, 37]. Furthermore, it is important to emphasize the value of supportive and responsive leadership with regard to confronting moral distress, as it has been shown to reduce moral distress in the context of long-term care [15].

### Strengths and limitations

We consider it a strength that our study provides a real picture of the variation in Norwegian long-term care facilities. This variation is due to the inclusion of nursing staff members with different levels of education working in diverse units: sheltered units for people with dementia and short- and long-term units from four long-term care facilities. In addition, we believe that the close cooperation of the research team and its reflection on the data throughout all stages of the research process are strengths of this study.

This study has limitations, and part of the recruitment and information gathering process may have some deficiencies. The management team in each long-term care facility was asked to recruit nursing staff members who might be interested in participation in the study and give them oral and written information about the project. Regardless of this process, it seemed that some of the informants had not received sufficient information about the study and enough time to think things through and prepare for the interviews. In addition, recruitment may have been affected by the preferences of the management team. The management team members could have chosen informants that they considered suitable, and other informants who might have added important information may have been excluded. It can also be considered a weakness of the study that we did not ask questions about moral stress in the original study. Despite this, important data emerged on moral stress that can provide an important source of knowledge in the field.

## Conclusion

This study found that nursing staff members experience moral distress in situations in which care and treatment cause people with severe dementia to suffer at the end of life. Moral distress was related to institutional limitations such as time constraints and challenging prioritizations but was more often related to what Jameton describes as “conflict with others about values”. Moral distress was generated when the nursing staff members felt obligated to provide care and treatment to residents with severe dementia that conflicted with their own values and knowledge about good palliative care. The outcomes of moral distress may manifest internally or externally and constitute a threat to good-quality dementia care.

### Relevance for clinical practise

Our findings indicate that moral distress is prevalent among nursing staff members who provide palliative care in long-term care facilities, and interventions to prevent moral distress are therefore needed. Utilizing a collaborative and palliative care approach to dementia care involving all parties in decision making could improve end-of-life care for residents and reduce the experience of moral distress among nursing staff. Additionally, education interventions focused on improving nursing staff members’ skills in communication, ethical judgement and coping may be useful for preventing moral distress. Supportive and responsive leadership has also been shown to reduce moral distress.

Our findings indicate a need for further research on interventions that can support nursing staff members dealing with ethical conflicts when providing palliative care to residents with dementia. Intervention studies have been undertaken, and it is crucial to generate evidence about interventions that can prevent moral distress in dementia caregivers*.*

## Data Availability

The data sets generated and/or analysed during the current study are available from the corresponding author on reasonable request.

## Abbreviations

P: Participant(s)
LTCF: Long-term care facility
RN: Registered nurse
EN: Enrolled nurse
GP: General practitioner
